# Control of Low-Density Lipoprotein Cholesterol in Secondary Prevention of Coronary Artery Disease in Real-Life Practice: The DAUSSET Study in French Cardiologists

**DOI:** 10.3390/jcm10245938

**Published:** 2021-12-17

**Authors:** Jean Ferrières, François Roubille, Michel Farnier, Patrick Jourdain, Denis Angoulvant, Franck Boccara, Nicolas Danchin

**Affiliations:** 1 Department of Cardiology, Toulouse Rangueil University Hospital, INSERM UMR 1295, Toulouse University School of Medicine, 31059 Toulouse, France; 2 PhyMedExp, INSERM, CNRS, Cardiology Department, INI-CRT, CHU de Montpellier, Université de Montpellier, 34090 Montpellier, France; francois.roubille@gmail.com; 3 Equipe PEC2, EA 7460, Service de Cardiologie, CHU Dijon Bourgogne, Université de Bourgogne Franche-Comté, 21000 Dijon, France; farnier.michel@orange.fr; 4 CHU Bicêtre AP-HP, Inserm U999, Université Paris-Saclay, 91190 Gif-sur-Yvette, France; patrick.jourdain@aphp.fr; 5 Service de Cardiologie, Hôpital Trousseau, CHRU de Tours, EA4245 T2i Faculté de médecine et Université de Tours, 37000 Tours, France; denis.angoulvant@univ-tours.fr; 6 GRC n°22, C^2^MV-Complications Cardiovasculaires et Métaboliques chez les Patients Vivant avec le Virus de L’immunodéficience Humaine, INSERM UMR_S 938, Centre de Recherche Saint-Antoine, Institut Hospitalo-Universitaire de Cardio-métabolisme et Nutrition (ICAN), Hôpital Saint-Antoine Service de Cardiologie, Assistance Publique-Hôpitaux de Paris, Sorbonne Université, 75012 Paris, France; franck.boccara@aphp.fr; 7 Département de Cardiologie, Hôpital Européen Georges Pompidou et Université de Paris, 75015 Paris, France; nicolasdanchin@yahoo.fr

**Keywords:** dyslipidemia, hypercholesterolemia, coronary artery disease, myocardial infarction, secondary prevention, guidelines adherence

## Abstract

Introduction: Patients with established coronary artery disease (CAD) are at very high risk for cardiovascular events. Methods: The DAUSSET study is a national, multicenter, non-interventional study that included very high-risk CAD patients followed by French cardiologists. It aimed to describe real-life clinical practices for low-density lipoprotein (LDL) cholesterol control in the secondary prevention of CAD. Results: A total of 912 patients (mean age, 65.4 years; men, 76.1%; myocardial infarction, 69.4%; first episode, 80.1%) were analyzed. The LDL cholesterol goal was 70 mg/dL in most cases (84.9%). The LDL cholesterol goal <70 mg/dL was achieved in 41.7% of patients. Of the 894 (98.0%) patients who received lipid-lowering therapy, 81.2% had been treated more intensively after the cardiac event, 27.0% had been treated less intensively and 13.1% had been maintained. Participating cardiologists were very satisfied or satisfied with treatment response in 72.6% of patients. Moderate satisfaction or dissatisfaction with lipid-lowering therapy was related to not achieving objectives (100%), treatment inefficacy (53.7%), treatment intolerance (23.4%) and poor adherence (12.3%). Conclusion: These real-world results show that lipid control in very high-risk patients remains insufficient. More than half of the patients did not achieve the LDL cholesterol goal. Prevention of cardiovascular events in these very high-risk patients could be further improved by better education and more intensive lipid-lowering therapy.

## 1. Introduction

Large epidemiological studies have shown a close and direct relationship between blood lipid levels and the risk of coronary artery disease (CAD) and stroke [1,2]. Hypercholesterolemia is a major contributor to the development of CAD and high levels of low-density lipoprotein cholesterol (LDL-C) are associated with an increased risk of CAD [3]. In a meta-analysis of 90,056 patients from 14 randomized statin trials, a 39 mg/dL (1 mmol/L) decrease in LDL-C levels in patients with CAD reduced the 5-year rate of major vascular events by approximately one fifth [4].

As a result, reducing levels of LDL-C has become a major objective of guidelines for the treatment and secondary prevention of atherosclerotic cardiovascular disease [2,5,6,7,8]. In patients at very high cardiovascular risk, the goal defined by the 2016 European guidelines was an LDL-C level <70 mg/dL (1.8 mmol/L) or at least a 50% reduction if the reference value was between 70 and 135 mg/dL [5]. In the 2019 ESC/EAS dyslipidemia guidelines, a target of 55 mg/dL LDL-C is recommended for all patients with very high-risk criteria and 40 mg/dL for patients with a second vascular event within 2 years [7].

Despite these recommendations, real-world studies have shown that only a small percentage of high-risk patients achieved lipid goals. For example, the EUROASPIRE IV survey conducted in 24 European countries showed that only 19.5% of patients with CAD had LDL-C levels <70 mg/dL [9]. In the first Dyslipidemia International Study (DYSIS), only 21.7% of very high-risk patients in European countries achieved their LDL-C goal [10,11]. In the DYSIS II study in seven European countries, LDL-C levels were <70 mg/dL in 28.3% of patients with stable CAD and 15.7% in patients who had been hospitalized with acute coronary syndrome (ACS) [12]. More recently, the ICLPS study in non-Western European countries outside the USA and Canada showed that only 28.5% of the very high-risk population achieved the recommended goal [13].

Cardiologists are on the forefront of treating patients. Various factors may affect the efficacy of treatment, such as poor adherence, side effects related to lipid-lowering treatment (LLT), insufficient response to treatment, drug interactions or associated comorbidities, but also factors related to the patient’s lifestyle or socio-economic conditions. The present study was designed to evaluate the practices of cardiologists, in real-world conditions, in the management of LDL-C risk in patients diagnosed with CAD. The primary objective of the study was to describe the current practice of cardiologists in managing lipid risk in patients with established CAD (secondary prevention). For this purpose, we assessed the rate of patients with an established diagnosis of CAD who achieved the LDL-C goal <70 mg/dL and the rates of treatment changes.

## 2. Materials and Methods

### 2.1. Study Design

The “observatoire national pour l’évaluation Des prAtiques des cardiologUes danS la priSe en charge du risquE lipidique en prévenTion secondaire (DAUSSET)” is a national, multicenter, non-interventional study conducted among French cardiologists. The objective of the study was to describe, in a real-life setting, the clinical practices for LDL-C risk control in secondary prevention of CAD.

The study conformed to the principles of the Declaration of Helsinki and Good Clinical Practice Guidelines. It was approved by the Ethics Committee “CPP NORD-OUEST II” (ID-RCB: 2017-A00280-53).

Investigators from a representative random sample of cardiologists in metropolitan France were asked to consecutively include patients who met the inclusion criteria.

### 2.2. Patients

Adult patients were included if they had established CAD treated for secondary prevention. The diagnosis of CAD comprised documented acute coronary syndrome: ST-segment elevation myocardial infarction (STEMI), non-ST-segment elevation myocardial infarction (NSTEMI), unstable angina, silent ischemia or stable angina documented by coronary angiography (stenosis >50%).

Patients had to be seen by the cardiologist more than 3 months but less than 3 years after the onset of the index coronary event, defined as the last documented ACS or diagnosis of CAD (coronary stenosis >50% in patients with stable angina or silent myocardial ischemia). Lipid tests performed on the following dates were recorded: before the index coronary event (diagnosis of CAD or ACS, whichever was the latest), at the time of the index coronary event (within 7 days of the event) and within 3 months before study entry. Only lipid tests at the time of the index coronary event were mandatory.

Patients with secondary dyslipidemia (uncontrolled hypothyroidism or nephrotic syndrome) and those participating in a clinical study that might alter the lipid profile (involving the use of lipid-lowering drugs) were not eligible to participate in the study.

### 2.3. Study Objectives

The primary objective of the study was to describe the current practice of cardiologists in managing lipid risk in patients with established CAD (secondary prevention).

The main secondary objectives were to describe the study population (demographics, cardiovascular risk factors) and patient care pathway; to assess the prevalence of possible familial hypercholesterolemia in the current clinical practice of cardiologists; to describe the therapeutic goals of cardiologists in managing lipid risk in the patient population (target level of LDL-C defined for the patient); to describe the therapeutic strategies employed by cardiologists in lipid risk management in secondary prevention; and to describe patient adherence to LLT.

### 2.4. Data Collection

Study data were collected by the physicians in a case report form during the visit from medical files, clinical examination and patient questioning. Patient data included sociodemographic data (age, gender, occupation, physical activity level); clinical examination at inclusion (weight, height, blood pressure, heart rate, known clinical signs suggesting familial hypercholesterolemia); personal cardiovascular risk factors (high blood pressure, known hypercholesterolemia before the event, microalbuminuria >30 mg/24 h, type 2 diabetes, smoking, sedentary lifestyle, rheumatoid arthritis, depression, HIV); description of the index coronary event, i.e., diagnosis of CAD or last ACS, regardless of which event occurred last; type (ST-segment elevation MI (STEMI), non-ST-segment elevation MI (NSTEMI), unstable angina, diagnosis of coronary disease for patients with stable angina or silent myocardial ischemia); territory; previous LLT; result of last available coronary angiography; revascularization procedure during ACS; acute heart failure; immediate post-ACS treatment (LLT, concomitant treatment); personal history of CAD prior to the index cardiac event; personal history of other cardiovascular disease; family history of cardiovascular disease; patient care pathway from the onset of cardiologic follow-up (referral to investigator, follow-up by investigator since the index coronary event or before cardiac rehabilitation program at the time of the index coronary event, adherence to lifestyle recommendations); therapeutic objectives set for the patient by the cardiologist (LDL-C goal); compliance and satisfaction of the physician with the response to treatment; and, if applicable, reasons for failure to achieve the therapeutic objective.

### 2.5. Statistical Analysis

The analyses were essentially descriptive, and no formal hypotheses were tested. We calculated that describing a 50% response rate with sufficient precision (3%) required a sample size of at least 1000 patients.

The primary endpoints were the rate of patients with an established diagnosis of CAD who achieved the LDL-C goal <70 mg/dL; the rate of patients whose treatment was stepped up (initiation of statin therapy, increase in the current statin dose, switch to a more potent statin, initiation of a new compound, e.g., ezetimibe); the rate of patients whose treatment was reduced (discontinuation or reduction of statin therapy or switch to a less potent statin therapy); and the rate of patients maintained on LLT overall.

Statistical analyses were performed using SAS software version 9.3 (SAS Institute, Inc., Cary, North Carolina, USA).

## 3. Results

### 3.1. Characteristics of Participating Cardiologists

A total of 77 cardiologists practicing in public or private hospitals (57.1%), with private practice (23.4%) or with mixed practice (19.5%) enrolled at least one patient. They were distributed throughout the national territory (46 out of the 96 French metropolitan departments). They had a median of 37 patients per month (range, 20–80) for secondary prevention.

### 3.2. Patient Characteristics and Index Cardiac Event

From July 2017 to October 2018, 1005 patients were selected, 93 of whom were not eligible. The analysis population was composed of 912 patients with a mean (SD) age of 65.4 (11.8) years; the majority were male (76.1%) and the mean (SD) body mass index was 27.1 (4.4) kg/m2 (Table 1). The main risk factors reported were treated hypertension (49.0%), known hypercholesterolemia before the cardiac event (46.7%), smoking (40.7%), sedentary lifestyle (37.7%) and type 2 diabetes (21.5%). A family history of premature cardiovascular disease was reported in 14.3% of male and 6.1% of female family members. The most common cardiovascular history was coronary revascularization (17.8%) and myocardial infarction (12.5%) (Table 1).

The index event occurred a mean (SD) of 16.8 (9.2) months prior to inclusion (Table 2). It was the first episode for 80.1% of patients. The most frequent index events were STEMI (39.6%) and NSTEMI (29.8%); diagnosis of coronary disease and unstable angina accounted for 16.1% and 14.5% of cases, respectively. The main locations of index events were anterior (42.3%) and inferior (29.8%). Patients had received prior lipid-lowering therapy in 39.4% of cases.

Coronary angiography most frequently revealed single-vessel disease (40.2%) and two-vessel disease (35.6%) (Table 2). Most patients had undergone a percutaneous coronary intervention with stent implantation (83.6%); there was no revascularization procedure in only 7.5% of patients.

At discharge, almost all patients received LLT (97.9%), most frequently at high intensity (76.9%).

### 3.3. Lipid Assessments

**Figure 1:**
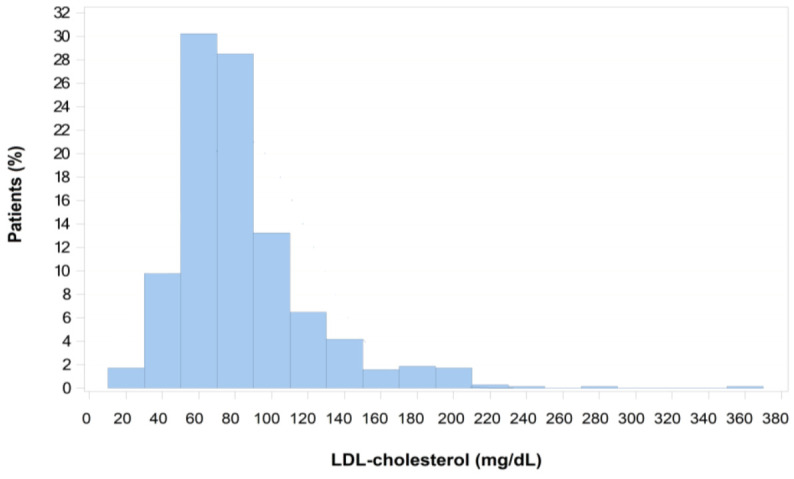
LDL-cholesterol at inclusion.

Lipid assessments before the index event were reported for only a small number of patients. However, the values reported for lipid work-up before and just after the index cardiac event were comparable (Table 3 and Figure 1). From the index date to study inclusion, mean total cholesterol values improved from 191 to 157 mg/dL and mean LDL-C values from 118 to 83 mg/dL. At inclusion, 22.0% of patients had LDL-C >100 mg/dL.

### 3.4. Follow-Up by Cardiologist after Index Cardiac Event and LDL-C Goal

In 66.8% of cases, participating cardiologists followed the patient before the index cardiac event for a mean (SD) duration of 8.1 (7.0) years (Table 4). Patients were referred to the participating cardiologists either directly after the cardiac event (39.5%), by the attending physician (36.1%) or by the hospital or clinic (24.3%). Patients attended a cardiac rehabilitation program primarily at a rehabilitation center (80.3%). Patients were considered adherent to a healthy lifestyle and treatment in 70.9% and 93.5% of cases, respectively.

The LDL-C goal defined by the cardiologist was 70 mg/dL in most cases (84.9%). This goal was communicated to the patient and attending physician in 88.0% and 80.6% of cases, respectively.

The participating cardiologists were very satisfied or satisfied with the response to treatment in 72.6% of cases. When the participating cardiologists were only moderately satisfied or dissatisfied, the main reasons were: objective not reached (100%), treatment inefficacy (53.7%), treatment intolerance (23.4%) and poor compliance to treatment (12.3%).

LDL-C <70 mg/dL was achieved in 41.7% of patients at inclusion. Of the 894 (98.0%) patients with LLT, treatment had been intensified in 81.2%, decreased in 27.0% and maintained in 13.1% since the index cardiac event (Table 5).

Achievement of the LDL-C goal was higher when the duration from the index event was ≤12 months (45.5%; 90/308) compared with >12 months (40.2%; 200/497).

Subgroup analyses indicated that LLT after the index cardiac event was more frequently intensified (95.3%) and LDL-C <70 mg/dL was more frequently achieved (45.4%) in patients without LLT before the index cardiac event; these percentages were 35.7% and 59.6% in patients with LLT before the index cardiac event, respectively.

## 4. Discussion

The characteristics of the study patients with established CAD, treated for secondary prevention and therefore at very high cardiovascular risk, were as expected (mean age, 65.4 years; men, 76.1%; myocardial infarction, 69.4%; history of hypertension, 49.0%; hypercholesterolemia, 46.7%; type II diabetes, 21.5%). Clinical signs of familial hypercholesterolemia were observed in 0.9% of patients. Comparable patient characteristics were reported in French patients with ACS in the DYSIS II study performed in 2013–2014 [14].

Patients received LLT at discharge in 97.9% of cases, most often at high intensity (76.9%); at inclusion in the study, 98.0% of patients were on LLT. At follow-up, therapy had been intensified in 81.2% of patients since the cardiovascular event. These data suggest that cardiologists followed guidelines and recommendations by prescribing higher doses of statins in very high-risk patients [5]. Indeed, in addition to lifestyle modifications, prescribing a statin at the highest recommended dose or the highest tolerable dose to achieve the LDL-C goal is the first choice [6,7]. Ezetimibe is recommended for patients who are intolerant to statins or who do not achieve LDL-C goal with statin monotherapy [6,7].

The LDL-C goal was set by the cardiologist at 70 mg/dL for 84.9% of patients and at 70–100 mg/dL for 14.4%. According to the 2016 ESC/EAS guidelines that were ongoing at the time of the study, an LDL-C level <70 mg/dL or a reduction of at least 50% if the baseline value was between 70 and 135 mg/dL was recommended for patients at very high cardiovascular risk [5]. These results indicate that almost all patients had LDL-C goals set as recommended [5].

From index cardiac event to study inclusion, mean total cholesterol and LDL-C values decreased from 191 to 157 mg/dL and from 118 to 83 mg/dL, respectively. An LDL-C goal of <70 mg/dL was achieved in 41.7% of patients. These results on the achievement of the LDL-C goal can be compared with those of other studies. In the subgroup of French patients hospitalized with an ACS in the DYSIS II study, statins were used in 96.6% of patients at discharge and in 95.1% of patients at 120-day follow-up [14]. At that time, 50.6% (80/158) of patients with available data achieved the LDL-C goal. The longer duration from index cardiovascular event to inclusion (16.8 vs. 4 months) may explain the slightly lower achievement of LDL-C goal in our study. In the first DYSIS study performed in 2008–2009, 15.3% of the 1470 patients with CAD enrolled in France achieved the LDL-C goal <70 mg/dL [15]. In the recent International Cholesterol Management Practice Study (ICLPS) conducted in 18 countries outside Western Europe, the USA and Canada, 28.5% of the 4882 patients with very high risk had LDL-C <70 mg/dL [13]. Overall, these results indicate that lipid control in very high-risk patients remains insufficient despite the availability of LLT. Even in Western countries, the achievement of LDL-C goals remains suboptimal [14].

LLT before the index cardiac event was reported in 39.4% of patients. In the subgroup of patients without LLT prior to the index cardiac event, LLT was more frequently intensified after the index cardiac event (95.3% vs. 45.4%), as expected. In these patients, the LDL-C goal was achieved more frequently than in patients with prior LLT (45.4% vs. 35.7%). Comparable results were obtained in DYSIS II Europe: 4 months after hospitalization for ACS, LDL-C levels <70 mg/dL were achieved in 41.5% and 30.9% of patients without LLT or with LLT prior to ACS, respectively [12]. Presumably, regular refills of the same prescription or experience with previous adverse events lead to maintenance of the same dose in patients with previous LLT. On the other hand, patients who have never been on long-term therapy seem to be more willing to intensify it, thus achieving better LDL-C control rates.

Although only 41.7% of patients achieved LDL-C goals in our study, 42.8% of cardiologists were very satisfied and 29.8% were satisfied with the response to therapy. Significant improvement in LDL-C without achieving the goal is a possible explanation for these satisfaction rates, as well as considering patients “close to goal” as an acceptable result. When cardiologists were moderately satisfied/dissatisfied, the main reasons were not achieving objective and ineffective treatment. Treatment intolerance and poor compliance were less frequently reported.

This study has the limitations of an observational study. In addition, it was performed in France and its generalization to other countries is uncertain. Some data were missing, including LDL-C values before the index cardiovascular event, and, to a lesser extent, at study inclusion (only one lipid work-up after the index cardiac event was mandatory). Participating cardiologists were randomly selected throughout the French metropolitan territory and were asked to enroll patients consecutively. However, not all French departments were represented and some unknown biases remain possible. Therefore, the cohort could not be fully representative of the French population at very high cardiovascular risk treated for secondary prevention of CAD. Two thirds of the patients had already been followed by the study investigator before the index cardiac event for a mean duration of 8.1 years. Thus, this population appeared to be rather medically privileged and the estimate of the rate of achievement of the LDL-C goal in the general population was probably overestimated. Another limitation of the study is the absence of detailed analysis of LLT, such as the achievement of LDL-C according to monotherapy, non-statin LLT or combination.

## 5. Conclusions

These real-world results suggest that lipid control in very high-risk patients remains insufficient. More than half of the patients did not achieve the LDL-C goals. Prevention of cardiovascular events in these very high-risk patients could be further improved by better education and more intensive LLT.

## Figures and Tables

**Table 1:** Characteristics of very high-risk CAD patients included in the study.

	Number of Patients Evaluated	Analysis Population (*n* = 912)
Age, years, mean (SD)	912	65.4 (11.8)
Gender, n (%)		
Male	912	694 (76.1)
Female	912	218 (23.9)
Body mass index, kg/m^2^, mean (SD)	889	27.1 (4.4)
Systolic blood pressure, mmHg, mean (SD)	898	133.1 (17.4)
Diastolic blood pressure, mmHg, mean (SD)	898	76.4 (10.2)
Heart rate, beats/min, mean (SD)	889	66.1 (11.1)
Clinical signs of familial hypercholesterolemia, n (%)	857	8 (0.9)
Risk factors, n (%)		
Treated hypertension	912	447 (49.0)
Known hypercholesterolemia before cardiac event	912	426 (46.7)
Smoking	887	391 (44.1)
Sedentary lifestyle	897	338 (37.7)
Type 2 diabetes	912	196 (21.5)
Depressive disorder	909	56 (6.2)
Microalbuminuria >30 mg/24 h	422	15 (3.6)
Untreated hypertension	912	24 (2.6)
HIV infection	863	15 (1.7)
Rheumatoid arthritis	906	5 (0.6)
Patient cardiovascular history, n (%)		
Coronary revascularization	912	162 (17.8)
Myocardial infarction	912	114 (12.5)
Unstable angina	912	67 (7.3)
Peripheral artery disease	909	58 (6.4)
Ischemic stroke	909	52 (5.7)
Stable angina	912	50 (5.5)
Heart failure	910	38 (4.2)
Silent myocardial infarction	912	11 (1.2)
Hemorrhagic stroke	910	2 (0.2)
Family history of premature cardiovascular disease, n (%)		
Male	781	112 (14.3)
Female	781	48 (6.1)

**Table 2:** Characteristics of the index cardiac event of the very high-risk CAD study patients.

	Number of Patients Evaluated	Analysis Population (*n* = 912)
Age at the index event, years, mean (SD)	912	64.1 (11.8)
Time between index event and inclusion, months, mean (SD)	912	16.8 (9.2)
Type of occurrence, n (%)		
First episode	911	730 (80.1)
Recurrence	911	181 (19.9)
Type of event, n (%)		
ST-segment elevation myocardial infarction	912	361 (39.6)
non-ST-segment elevation myocardial infarction	912	272 (29.8)
Coronary disease diagnosis	912	147 (16.1)
Unstable angina	912	132 (14.5)
Main locations of the index event, n (%) a		
Anterior	898	380 (42.3)
Inferior	898	268 (29.8)
Lateral	898	103 (11.5)
Previous lipid-lowering therapy, n (%)	884	348 (39.4)
Results of coronary angiography, n (%)		
Single-vessel disease	909	365 (40.2)
Two-vessel disease	909	324 (35.6)
Three-vessel disease	909	198 (21.8)
Left main artery	909	4 (0.4)
Single-vessel disease and left main artery	909	1 (0.1)
Two-vessel disease and left main artery	909	6 (0.7)
Three-vessel disease and left main artery	909	11 (1.2)
Revascularization procedure, n (%)		
None	912	68 (7.5)
Angioplasty with stent	912	762 (83.6)
Angioplasty without stent	912	21 (2.3)
Angioplasty (no information on stent)	912	2 (0.2)
Coronary bypass surgery	912	59 (6.5)
Acute heart failure during acute phase, n (%)	904	79 (8.7)
Lipid-lowering therapy at discharge, n (%) ^a^	912	893 (97.9)
Low intensity	893	38 (4.3)
Moderate intensity	893	168 (18.8)
High intensity	893	687 (76.9)
Associated treatment at discharge, n (%)	912	911 (99.9)
Beta blockers	910	802 (88.1)
Renin-angiotensin system blockers	910	717 (78.8)
Calcium channel inhibitors	907	121 (13.3)
Nitroglycerin	902	103 (11.4)

^a^ Low-intensity lipid-lowering therapy lowers LDL-C by <30% on average, moderate intensity therapy by approximately 30% to 50% on average and high intensity therapy by approximately ≥50% on average.

**Table 3:** Lipid assessment of very high-risk CAD patients before and after the index cardiac event.

	Number of Patients Evaluated	Analysis Population (*n* = 912)
Before the index event		
Lipid-lowering treatment, n (%)	296	143 (48.3)
Total cholesterol, mg/dL, mean (SD)	134	196 (55)
LDL-cholesterol, mg/dL, mean (SD)	143	121 (48)
HDL-cholesterol, mg/dL, mean (SD)	136	49 (19)
Triglycerides, mg/dL, mean (SD)	139	144 (82)
After the index cardiac event (within 7 days)		
Lipid-lowering treatment at discharge, n (%)	912	893 (97.9)
Total cholesterol, mg/dL, mean (SD)	901	191 (55)
LDL-cholesterol, mg/dL, mean (SD)	895	118 (047)
HDL-cholesterol, mg/dL, mean (SD)	910	45 (16)
Triglycerides, mg/dL, mean (SD)	908	147 (95)
At study inclusion (within 3 months)		
Lipid-lowering treatment, n (%)	912	894 (98.0)
Total cholesterol, mg/dL, mean (SD)	689	157 (45)
LDL-cholesterol, mg/dL, mean (SD)	695	83 (37)
LDL-cholesterol >100 mg/dL, n (%)	695	153 (22.0)
HDL-cholesterol, mg/dL, mean (SD)	695	48 (16)
Triglycerides, mg/dL, mean (SD)	689	131 (96)

**Table 4:** Patient care after index cardiac event and LDL-C goal.

	Number of Patients Evaluated	Analysis Population (*n* = 912)
Follow-up by the investigator before index cardiac event, n (%)	911	609 (66.8)
Duration of follow-up, years, mean (SD)	302	8.1 (7.0)
Referral to participating cardiologist for the first time, n (%)		
Hospital or clinic	912	222 (24.3)
Directly after cardiac event	912	360 (39.5)
Attending physician	912	329 (36.1)
Other	912	35 (3.8)
Cardiac rehabilitation program, n (%)	900	407 (45.2)
In hospital	407	87 (21.4)
In rehabilitation center	407	327 (80.3)
In hearth and health club	407	1 (0.2)
Compliance with healthy lifestyle, n (%)	911	646 (70.9)
Compliance with treatment, n (%)	898	840 (93.5)
Target LDL-C, mg/dL, n (%)		
50–70	912	6 (0.7)
70	912	774 (84.9)
70–100	912	55 (6.0)
100	912	77 (8.4)
Target LDL-C communicated to patient, n (%)	908	799 (88.0)
Target LDL-C communicated to attending physician, n (%)	895	721 (80.6)
Satisfaction of cardiologist for treatment response, n (%)		
Very satisfied	890	381 (42.8)
Satisfied	890	265 (29.8)
Moderately satisfied	890	160 (18.0)
Not at all satisfied	890	84 (9.4)
Reasons for moderate satisfaction/dissatisfaction, n (%) ^a^		
Objective not reached	244	244 (100)
Treatment inefficacy	244	131 (53.7)
Treatment intolerance	244	57 (23.4)
Poor treatment compliance	244	30 (12.3)
Treatment refusal	244	6 (2.5)
Rare dyslipidemia	244	1 (0.4)
Other reason	244	48 (19.7)

^a^ More than one answer was possible.

**Table 5:** Primary endpoints: achievement of target LDL-C <70 mg/dL and changes in lipid-lowering treatment.

	Number of Patients Evaluated	Analysis Population (*n* = 912)
LDL-C target achieved (<70 mg/dL), n (%)	695	290 (41.7)
Patients with lipid-lowering treatment, n (%) ^a^	912	894 (98.0)
Therapy intensification	894	726 (81.2)
Decrease in therapy	891	241 (27.0)
Lipid-lowering treatment maintained	894	117 (13.1)
Patients with no lipid-lowering treatment, n (%)	912	18 (2.0)

^a^ Patients could be counted both in “therapy intensification” class and in “decrease in therapy” class; patients in the “lipid-lowering treatment maintained” class were counted only once.

## Data Availability

Data are available from the corresponding author upon reasonable request.
